# A Bayesian spatio-temporal model for forecasting the prevalence of antibodies to *Borrelia burgdorferi*, causative agent of Lyme disease, in domestic dogs within the contiguous United States

**DOI:** 10.1371/journal.pone.0174428

**Published:** 2017-05-04

**Authors:** Stella C. Watson, Yan Liu, Robert B. Lund, Jenna R. Gettings, Shila K. Nordone, Christopher S. McMahan, Michael J. Yabsley

**Affiliations:** 1 Department of Mathematical Sciences, Clemson University, Clemson, SC, United States of America; 2 Department of Molecular and Biomedical Sciences, Comparative Medicine Institute, North Carolina State University, College of Veterinary Medicine, Raleigh, NC, United States of America; 3 Southeastern Cooperative Wildlife Disease Study, Department of Population Health, College of Veterinary Medicine, University of Georgia, Athens, GA, United States of America; 4 Warnell School of Forestry and Natural Resources, University of Georgia, Athens, GA, United States of America; Cary Institute of Ecosystem Studies, UNITED STATES

## Abstract

This paper models the prevalence of antibodies to *Borrelia burgdorferi* in domestic dogs in the United States using climate, geographic, and societal factors. We then use this model to forecast the prevalence of antibodies to *B. burgdorferi* in dogs for 2016. The data available for this study consists of 11,937,925 *B. burgdorferi* serologic test results collected at the county level within the 48 contiguous United States from 2011-2015. Using the serologic data, a baseline *B. burgdorferi* antibody prevalence map was constructed through the use of spatial smoothing techniques after temporal aggregation; i.e., head-banging and Kriging. In addition, several covariates purported to be associated with *B. burgdorferi* prevalence were collected on the same spatio-temporal granularity, and include forestation, elevation, water coverage, temperature, relative humidity, precipitation, population density, and median household income. A Bayesian spatio-temporal conditional autoregressive (CAR) model was used to analyze these data, for the purposes of identifying significant risk factors and for constructing disease forecasts. The fidelity of the forecasting technique was assessed using historical data, and a Lyme disease forecast for dogs in 2016 was constructed. The correlation between the county level model and baseline *B. burgdorferi* antibody prevalence estimates from 2011 to 2015 is 0.894, illustrating that the Bayesian spatio-temporal CAR model provides a good fit to these data. The fidelity of the forecasting technique was assessed in the usual fashion; i.e., the 2011-2014 data was used to forecast the 2015 county level prevalence, with comparisons between observed and predicted being made. The weighted (to acknowledge sample size) correlation between 2015 county level observed prevalence and 2015 forecasted prevalence is 0.978. A forecast for the prevalence of *B. burgdorferi* antibodies in domestic dogs in 2016 is also provided. The forecast presented from this model can be used to alert veterinarians in areas likely to see above average *B. burgdorferi* antibody prevalence in dogs in the upcoming year. In addition, because dogs and humans can be exposed to ticks in similar habitats, these data may ultimately prove useful in predicting areas where human Lyme disease risk may emerge.

## Introduction

Lyme disease, the most common zoonotic tick-borne disease in the United States and Europe [1], is caused by bacterial spirochetes from the *Borrelia burgdorferi* sensu lato complex, and is transmitted by ticks in the genus *Ixodes* [2, 3]. *Borrelia burgdorferi* can infect and cause acute and/or chronic Lyme disease in both humans and dogs. Clinically, there are similarities in disease presentation, and diagnosis and treatment follow similar guidelines. In 2014, the Centers for Disease Control and Prevention (CDC) reported 30,000 confirmed human Lyme disease cases, with an estimated 329,000 additional probable cases based on medical claims information from a large insurance database [4, 5]. The Companion Animal Parasite Council (CAPC) reported 250,880 dogs, out of 4 million dogs tested, were positive for antibodies to *B. burgdorferi* in 2015 [6]. While the cost of Lyme disease diagnosis and treatment in dogs is not documented, the cost to the US healthcare system for care of humans with Lyme disease is substantial: treatment of Lyme disease and post-treatment Lyme disease syndrome (PTLDS) cost between $712 million and $1.3 billion annually [7]. The incidence of disease has been increasing steadily over the last decade [8], and as the number of cases increase, the economic impact of Lyme disease is expected to increase as well.

Clinical Lyme disease manifests similarly in people and dogs, with infection most commonly causing transient fever, anorexia, and arthritis [9, 10]. Early erythema migrans lesions have been observed in up to 75% of human patients [11], but are no longer considered pathognomonic for Lyme disease [9, 11]. Chronic disseminated disease in humans may lead to musculoskeletal, neurologic, dermatologic, and rarely cardiac disease [12–16]. Chronic disease in dogs is more often associated with arthropathy but case reports of renal, neurologic, cardiac, and dermatologic disease exist [10, 17, 18]. Time to the onset of disease after infection, the incubation period, differs between dogs and humans. Dogs have been reported to have an extended two to five month incubation period before becoming symptomatic [10], in contrast to three to 30 days in humans [14]. The first signs of clinical disease in dogs are non-specific, including fever, general malaise, lameness, and swelling of local lymph nodes. These symptoms are likely to be overlooked by dog owners because they are transient, lasting only a few days [19]. Detecting the later stages of disease require recognition of pain, however, a standardized protocol for pain assessment in veterinary species is lacking [20–22] and mainly relies on dog owners to report disease symptoms. The assessment of pain in dogs can be difficult as they cannot self-report and is often reported by the owner as lethargy, decreased activity, or difficulty getting up, walking, or navigating stairs. It is often not until the dogs exhibit the characteristic shifting leg lameness several months after infection that owners note any abnormalities. Finally, Lyme disease is most commonly diagnosed by measurement of antibodies specific for *B. burgdorferi* (termed seropositivity). In humans, seropositivity is assessed in a two-step process involving an enzyme-linked immunosorbent assay (ELISA) and Western blot analysis of serum [23, 24]. Veterinary wellness exams commonly include annual screening for exposure to *B.burdgorferi*, as well as *Ehrlichia* spp., *Anaplasma* spp., and *Dirofilaria immitis* (etiologic agent of Heartworm disease) using a rapid, in-house ELISA platform (SNAP^®^3Dx^®^, SNAP^®^4Dx^®^ and SNAP^®^4Dx^®^ Plus, IDEXX Laboratories, Inc.). In this point-of-care assay, seropositivity against *B.burgdorferi* is established using the late phase C6 antigen that is indicative of disseminated disease [25–29]. Specifically in dogs, seroconversion to *B. burgdorferi* antigen C6 can occur as early as 3-4 weeks post-infection when bacterial burden is high [29]. Multiplex assays suggest antibodies to OspC, OspF, and C6 are characteristic of the intermediate stage of infection (at least 3-4 weeks post-infection), while antibodies to OspF and C6, in the absence of antibodies to OspC, are suggestive of late infection stage [29]. Data from point-of-care tests for C6 seropositivity have been used to develop distribution maps of *B. burgdorferi* seroprevalence in domestic dogs throughout the United States [30, 31].

In the United States, *B. burgdorferi* is transmitted by *Ixodes scapularis* on the East coast and Midwest and *Ixodes pacificus* on the West coast, while a variety of wildlife species (e.g., mice, squirrels, shrews) serve as reservoirs [1]. Although not infected with *B. burgdorferi*, deer and migratory birds play an important role in maintaining and transporting tick vectors [32, 33]. The risk of Lyme disease exposure is therefore related to abundance of a suitable reservoir and exposure to infected ticks. Overall, 96% of human Lyme disease cases reported by the CDC are from 14 states (Connecticut, Delaware, Maine, Massachusetts, Maryland, Minnesota, New Hampshire, New Jersey, New York, Pennsylvania, Rhode Island, Vermont, Virginia, and Wisconsin) [34]. Prevalence of exposure in dogs is similarly high in these states, although the range is expanded beyond the core areas of human cases within these states and into neighboring states such as Ohio, West Virginia and North Dakota [6]. While dogs show no age-specific epidemiological risk profile, children 5-9 years of age are at highest risk of Lyme disease, followed by adults aged 45-59 years [35]. This bimodal distribution of human risk is attributed to increased contact with the environment and exposure to infected ticks. Recent assessment of vector distribution identified a 45% increase in *Ixodes* spp. range over the last 18 years [36], with *I. scapularis* firmly established in twice as many counties in the North-Central and Northeastern US as previously believed. Importantly, the Northeastern and North-Central range of *I. scapularis* appears to be merging in the Ohio River Valley, forming a contiguous range of potential vector establishment.

The dynamic change in *Ixodes* spp. ranges suggests ongoing monitoring of these medically important vectors, while important, will be challenging and economically unfeasible on an annual basis. Understanding and forecasting spatial and temporal patterns of risk of exposure to *B. burgdorferi* is thus critical for targeting use of vaccines, preventives measures, and educational campaigns to best protect humans and veterinary species. Building on previously reported risk assessment and surveillance tools for vector-borne disease [37, 38], we now report on a novel predictive model of canine *B. burgdorferi* exposure using a Bayesian approach to factor assessment and forecasting. As previously described, Bayesian modeling offers a number of advantages over classical approaches [39, 40]. The probabilistic likelihood-based methods are highly flexible and are able to adapt to data availability constraints. These methods are also capable of assessing predictive significance of various covariate factors. The use of data augmentation Markov chain Monte Carlo (MCMC) methods to sample from a posterior distribution provides the opportunity to treat missing data, such as absence of serologic data from certain counties, as latent (missing) variables, even in large populations [41, 42]. Finally, a forecast of future seroprevalence, conditional on the past history of data, are easily constructed. The Bayesian methods capably quantify uncertainty both in terms of the potential stochasticity of the disease process and the model parameter estimates.

In what follows, eight factors previously purported to influence canine *B. burgdorferi* seroprevalence will be examined: annual precipitation, annual relative humidity, annual temperature, elevation, percentage forest coverage, percentage surface water coverage, population density, and median household income [43]. After assessment, a predictive model using the significant factors is developed, and annual *B. burgdorferi* seroprevalence forecasts are constructed. A comparison of actual versus predicted 2015 prevalence is conducted. Intended uses of annual *B. burgdorferi* seroprevalence forecasts for veterinary medicine include: 1) provision of an evidence-based tool used to encourage the year-round use of tick preventive to reduce exposure, and 2) promotion of annual use of diagnostic testing in areas where the disease is emerging. Finally, based on previous work using canine Lyme disease prevalence to assess human disease risk [17, 44], we highlight the use of canine disease forecast maps to inform risk for human disease in an effort to reduce the burden of human disease on the US healthcare system.

## Materials and methods

### Data structure

The observed data consist of test results from 11,937,925 *B. burgdorferi* ELISAs performed from 2011 to 2015 in the contiguous United States. The test detects antibodies produced in response to the C6 peptide found on the outer membrane protein, VlsE, of *B. burgdorferi* during infection [45]. Detection of antibodies does not indicate an active infection, as they will persist over time, even after the infection is resolved. Nor is this a quantitative test, so the time since infection is not known. The seroprevalence reported is a representation of the prevalence of dogs that have been exposed to *B. burgdorferi*, not the prevalence of clinically ill dogs. It is also important to note that the C6 ELISA does not detect antibodies to current vaccines [46], so vaccinated dogs will not test positive unless the vaccine has failed and the dogs experienced exposure resulting in disseminated infection. The data were provided by a commercial diagnostic laboratory available to veterinary clinics within the study area, IDEXX Laboratories, Inc., and included the county in which the testing clinic resides and the month in which the test was conducted [47]. The submitted samples represent a population of dogs provided veterinary care. No information was available on demographic details of the individuals tested, such as age, sex or breed of dog, nor the travel or testing history of the dog, or the reason why the tests were conducted. Overall, there were 759,103 positive tests, for an empirical prevalence of 0.0635. For the purposes of fitting the model, the data were aggregated by county and by year. Table 1 shows the eight considered factors thought to be associated with *B. burgdorferi* prevalence, along with the time period for which data on each factor is available and the level of geographic aggregation (county, state, etc.). For further discussion, including the source of each factor, see [43, 48].

**Table 1:** Factors purported to influence *B. burgdorferi* seroprevalence.

Factors and Notation	Data period	Scale
Annual temperature: *X*_*s*,1_(*t*)	1895–2015	Climate Division
Annual precipitation: *X*_*s*,2_(*t*)	1895–2015	Climate Division
Annual relative humidity: *X*_*s*,3_(*t*)	2006–2015	Climate Division
Elevation: *X*_*s*,4_(*t*)	2012	County
Percentage forest coverage: *X*_*s*,5_(*t*)	2012	County
Percentage surface water coverage: *X*_*s*,6_(*t*)	2010	County
Population density: *X*_*s*,7_(*t*)	2011–2014	County
Median household income: *X*_*s*,8_(*t*)	1997–2014	County

Note, *X*_*s*,*k*_(*t*) is used to denote the value of the *k*th factor in the *s*th county during the *t*th year.

**Fig 1:**
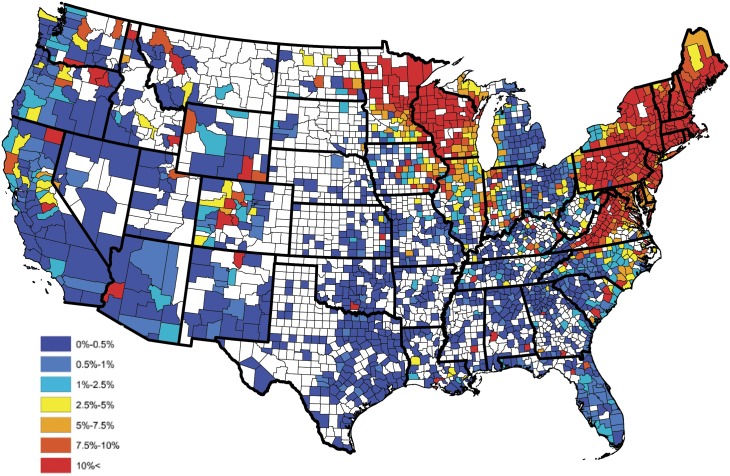
County level raw prevalences for *B. burgdorferi* antibodies in domestic dogs aggregated from 2011-2015.

**Fig 2:**
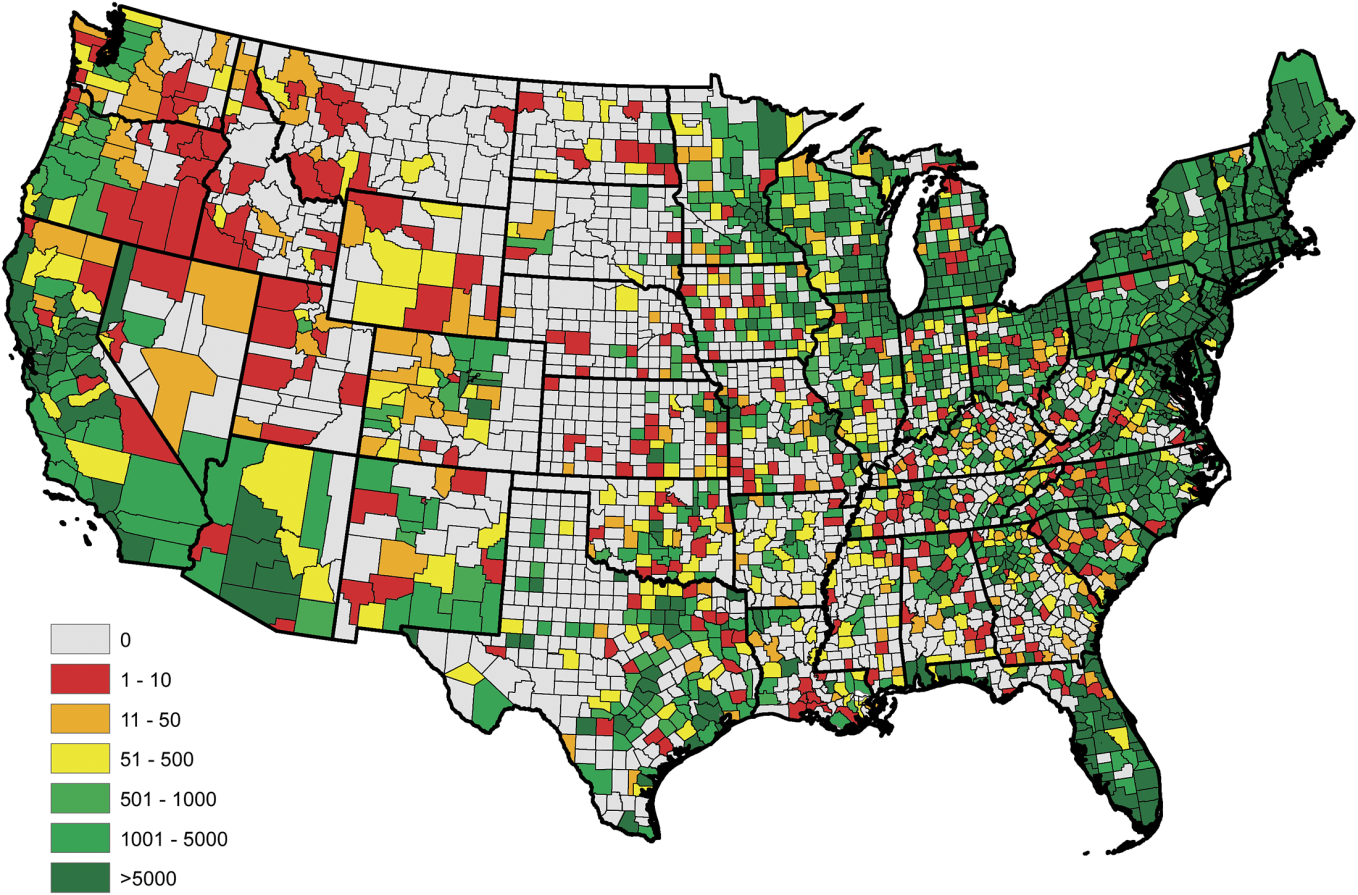
Total number of serologic test results for *B. burgdorferi* antibodies in domestic dogs collected within each county during the years of 2011-2015.

An empirical estimate of the prevalence within each county was constructed in the usual fashion after aggregating the serologic data over the available five year time span; i.e., these estimates were obtained as the proportion of positive tests observed within each reporting county. Figs 1 and 2 display the empirical prevalences and the total number of tests, respectively, within each reporting county. From Fig 1, one will note that there appears to be a great deal of positive spatial correlation. Moreover, in studies such as these temporal dependence is expected in the data. Thus, to provide an accurate analysis these spatio-temporal dependencies have to be accounted for in the model. Further, from Fig 2, one will note that some counties report relatively small number of tests, thus the interpretation of the aforementioned empirical estimates may be slightly misleading, if one does not consider this effect. For example, a county reporting only 20 test results, with 1 being positive, results in an empirical estimate of 5%, where as the same empirical estimate would be obtained for a county reporting 2000 test results, with 100 being positive. The salient point, more faith should be placed in the latter estimate, when compared to the former, since it is derived from more information.

**Fig 3:**
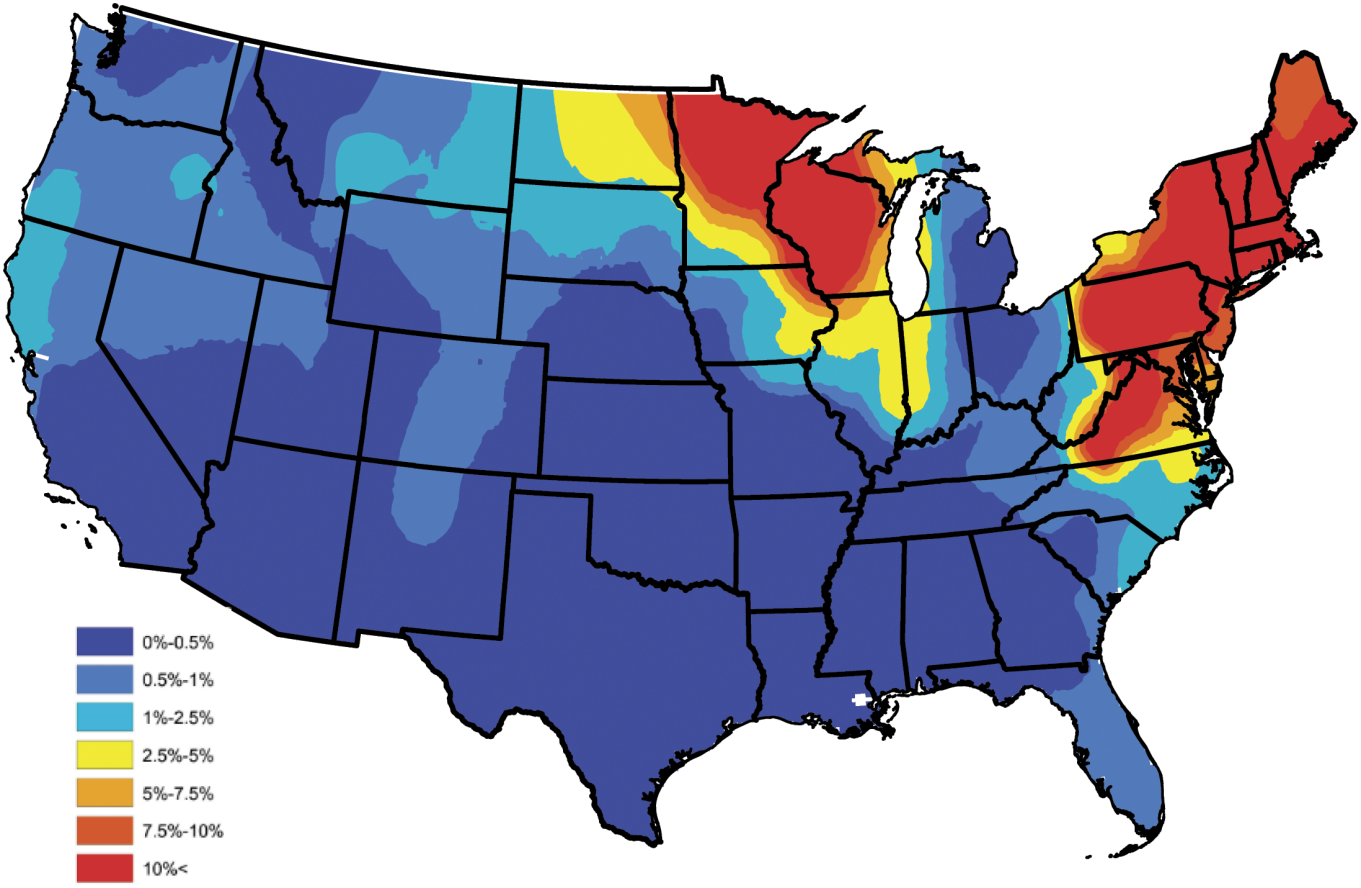
Head-banged baseline map showing *B. burgdorferi* antibody prevalences in domestic dogs for an average year during 2011-2015.

In order to construct a baseline *B. burgdorferi* antibody prevalence map, the empirical prevalences displayed in Fig 1 were subjected to an ensemble of spatial smoothing techniques. First, weighted head-banging was applied to the empirical prevalences, to acknowledge spatial correlation and diminish the influence of counties which reported only a few tests. For example, there were 57,785 test results reported in South Carolina during the year of 2015, of which 606 were positive, translating to an empirical prevalence estimate of 1%. Further, in Colleton county, South Carolina, only 13 test results were reported, of which 3 were positive, translating to an empirical prevalence estimate of 23%. Obviously, it is inappropriate to believe that the true prevalence is anywhere near as high as the empirical estimate tends to suggest in Colleton county, and that this effect would have been mitigated if more serologic data were available in this region. In the absence of additional serologic data, weighted head-banging provides a robust prevalence estimate at a given spatial location by combining over information that is available in near by areas, thus, for the most part circumventing the small sample size issue. This smoothing procedure used 45 triples and weighted the prevalence of each county proportional to the number of tests from that county. For further details on weighted head-banging and its uses within the context of disease mapping see [48]. Second, in order to render a spatially smooth and complete map Kriging was implemented to interpolate the prevalences of counties not reporting data. Kriging was implemented in ArcGIS using the default parameter settings [49]. Fig 3 provides the baseline *B. burgdorferi* antibody prevalence map resulting from this process, and should be thought of as a baseline for *B. burgdorferi* prevalence in domestic dogs within the contiguous United States. Tincubation conditions and hat is, this figure depicts the general spatial trend of *B. burgdorferi* prevalence in domestic dogs, and will serve as a device which allows one to assess the performance of Bayesian spatio-temporal model developed in the subsequent section, with respect to capturing these trends.

### Statistical model

This section describes the statistical model and the techniques used to fit it. The purpose of the model is twofold: to identify environmental and societal risk factors which are significantly associated with the prevalence of *B. burgdorferi* antibodies in dogs and to predict future trends in the prevalence of *B. burgdorferi* antibodies in dogs.

Let *Y*_*s*_(*t*) and *n*_*s*_(*t*) denote the number of positive and total tests conducted in county *s* during year *t*, respectively, for counties *s* ∈ {1, …, *S*} and years *t* ∈ {1, …, *T*}. It is important to note that these values are the raw counts available in the data set; i.e., they are not the post smoothed values resulting from the construction of the baseline map. The available serologic data exhibits both positive spatial and temporal correlation; that is, prevalences in adjacent counties or in successive years tend to have similar values. Thus, to account for these effects this analysis considers using a spatio-temporal model to analyze these data; for additional information about spatial and spatio-temporal models, see [50–56]. In particular, this paper uses a Bayesian hierarchical model, which models spatio-temporal dependence through the use of random effects. The details of the distribution of these random effects are described in totality below. In the considered model, the number of positive tests were assumed to follow a Poisson distribution, which is a common choice for modeling count data [52–55]. Under this specification, the number of tests which are serologically positive for *B. burgdorferi* antibodies in county *s* during year *t* (i.e., *Y*_*s*_(*t*)) is assumed to be distributed as

$$Y_{s}\left(t\right)|n_{s}\left(t\right),p_{s}\left(t\right)∼\text{Poisson}\left\{n_{s}\left(t\right)p_{s}\left(t\right)\right\},$$ (1)

$$\log\left\{p_{s}\left(t\right)\right\}=\beta_{0}+\sum_{k=1}^{p}\beta_{k}X_{s,k}\left(t\right)+\xi_{s}\left(t\right),$$ (2)

where log(⋅) denotes the natural logarithm, ′ denotes the transpose of a matrix (or vector), ∼ reads “has the distributional type,” and ∣ reads “given.” Thus, Eq (1) reads “*Y*_*s*_(*t*) given *n*_*s*_(*t*) and *p*_*s*_(*t*) follows a Poisson distribution with mean *n*_*s*_(*t*)*p*_*s*_(*t*).” Furthermore, **X**_*s*_(*t*) = (*X*_*s*,1_(*t*), …,*X*_*s*,*p*_(*t*))′ is a vector of covariate information for county *s* at time *t*, ***β*** = (*β*_0_, …, *β*_*p*_)′ is the corresponding vector of regression coefficients, and *p*_*s*_(*t*) is interpreted as the prevalence of *B. burgdorferi* antibodies in dogs residing in county *s* at time *t*. The random effects, *ξ*_*s*_(*t*), are used to account for the spatio-temporal dependence, so that the positive test counts (i.e., *Y*_*s*_(*t*)’s) are conditionally independent of each other given the total number of tests, the factor information, and the random effects. Note, this does not imply that the *Y*_*s*_(*t*)’s are independent across varying space *s* or time *t*, only that they are conditionally independent once the spatio-temporal correlation is accounted for through the random effects and the other covariate information.

Eq (2) specifies the relationship between the prevalence *p*_*s*_(*t*) and the covariate information **X**_*s*_(*t*) and the random effect *ξ*_*s*_(*t*). This specification is standard for Poisson regression models [52–55]. In general, different spatio-temporal models specify different structures for the {*ξ*_*s*_(*t*)}. By far, one of the most popular models for areal data is the conditional autoregresive (CAR) model, and it is the one used here [50]. To further expound on how the CAR model is used in this analysis, it is noted that both spatial and temporal correlation is accounted for through the following multivariate autoregressive model

$$\xi_{1}=\phi_{1},$$ (3)

$$\xi_{t}=\varphi \xi_{t-1}+\phi_{t},\text{for }t=2,\ldots ,T,$$ (4)

$$\phi_{t}∼\text{CAR}\left(\tau^{2};\rho \right),\text{for }t=1,\ldots ,T,$$ (5)

where ***ξ***_*t*_ = (*ξ*_1_(*t*), …, *ξ*_*S*_(*t*))′ and ***ϕ***_*t*_ = (*ϕ*_1_(*t*), …, *ϕ*_*S*_(*t*))′ are random vectors. Eq (5) specifies that ***ϕ***_*t*_ are independent and identically distributed random vectors whose distribution follows a CAR model [50, 51]. From Eq (4) it is apparent how the model accounts for temporal correlation. That is, in the multivariate autoregressive model depicted above, time-dependence is modeled through a temporal autoregressive model of order one (AR(1)), which is a staple among time series analysis [57]. Eq (4) relates year *t* to year *t* − 1. The parameter *φ* is the temporal correlation between consecutive years and lies within (−1,1). This ensures a causal and stationary solution to the time series model [57].

To examine the treatment of spatial correlation, let *ϕ* = (*ϕ*_1_, …, *ϕ*_*S*_)′ follow a CAR model, where for the ease of exposition the dependence on *t* has been suppressed, and it is noted that the subscripts correspond to the *S* spatial locations. There are several different kinds of CAR models. Usually, CAR models are defined by assigning a univariate distribution for each *ϕ*_*s*_, whose mean and variance depends on the spatial relationship between location *s* and the other locations. This paper uses the CAR model proposed in [51], which specifies the conditional distribution for each *ϕ*_*s*_ to be

$$\begin{array}{c} \phi_{s}|\phi_{-s},\tau^{2},\rho ,W∼N\left(\rho \frac{\sum_{s^{\prime }\neq s}w_{s,s^{\prime }}\phi_{s^{\prime }}}{\sum_{s^{\prime }\neq s}w_{s,s^{\prime }}},\;\;\frac{\tau^{2}}{\sum_{s^{\prime }\neq s}w_{s,s^{\prime }}}\right),\;\text{for }s=1,\ldots ,S. \end{array}$$ (6)

Here, ***ϕ***_−*s*_ = (*ϕ*_1_, …, *ϕ*_*s*−1_, *ϕ*_*s*+1_, …, *ϕ*_*S*_)′ is an *S* − 1 dimensional vector that includes every *ϕ*_*s*′_ except *ϕ*_*s*_. *N*(*μ*, *σ*^2^) denotes a normal distribution with mean *μ* and variance *σ*^2^. The *S* × *S* matrix **W** is defined by **W** = {*w*_*s*,*s*′_}, where *w*_*s*,*s*′_ = 1 if the *s*′th and *s*th counties are adjacent and *w*_*s*,*s*′_ = 0 otherwise.

The parameter *τ*^2^ in Eq (6) scales the variance structure. Moreover, the conditional variance of *ϕ*_*s*_, given ***ϕ***_−*s*_, is inversely proportional to the number of counties bordering it. That is, the *ϕ*_*s*_ for counties with more neighbors have a smaller overall variance. This agrees with intuition; if county *s* has many neighbors, the model has more information to use in estimating *ϕ*_*s*_, and thus the variance of *ϕ*_*s*_ should be smaller. In Eq (6), *ρ* ∈ [0, 1] controls the correlation between bordering counties. The conditional mean of *ϕ*_*s*_, given ***ϕ***_−*s*_, is the average of the *ϕ*_*s*′_ of the neighboring counties weighted by *ρ*. Thus, as *ρ* increases so does the degree of dependence between *ϕ*_*s*_ and the *ϕ*_*s*′_ of the neighboring counties.

Using Eq (6), it can be shown that the joint distribution of ***ϕ*** is given by the following multivariate normal distribution

$$\phi ∼N\left(0,\Gamma \right),\;\Gamma =\tau^{2}{\left(D-\rho W\right)}^{-1},$$

where **W** is the adjacency matrix described above and **D** is an *S* × *S* diagonal matrix whose *s*th diagonal element is the number of neighboring counties for county *s*.

The model is fit using Bayesian Markov Chain Monte Carlo (MCMC) techniques, with posterior samples being used to derive point estimates of the parameters (***β***, *φ*, *ρ*, and *τ*^2^). Thus, to complete the Bayesian model formulation, the following prior distributions are specified

$$\beta_{k}∼N\left(0,1000\right),\;\text{for}\;k=0,\ldots ,p;$$ (7)

φ∼Uniform(-1,1); (8)

ρ∼Uniform(0,1); (9)

$$\tau^{-2}∼\text{Gamma}\left(0.5,0.05\right).$$ (10)

In particular, a diffuse prior distribution is placed on *β*_*k*_, for *k* = 0, …, *p*. This specification permits the prior to exert little, if any, influence on the posterior distribution, thus allowing the data to primarily drive the analysis and subsequently the conclusions. Uninformative (flat) prior distributions are assigned for *φ* and *ρ*, for the same reasons. Here “uninformative” means that all possible values of the parameter have equal probability under the prior distribution. The prior for *τ*^−2^ is chosen as a conjugate prior. Here “conjugate” means that the posterior and prior distributions are from the same distributional family, which simplifies computation. A posterior sampling algorithm was developed, in the usual fashion, to sample all model parameters and random effects from the posterior distributions. This MCMC sampling algorithm uses a combination of Gibbs and Metropolis-Hastings steps. To complete model fitting, *Y*_*s*_(*t*) for counties not reporting test results were treated as latent variables, and were sampled along with the model parameters. The posterior sampling algorithm was conducted using code written in **R** and **C++**. For more information about Bayesian models and MCMC methods, see [39].

## Results

### Model assessment

This analysis first considered a full model consisting of all 8 climate, geographic, and societal factors. After fitting the full model, credible intervals were computed. Credible intervals are the Bayesian counterpart to frequentist confidence intervals. Table 2 provides the point estimates (i.e., the median of the posterior samples) of the regression coefficeints along with 98.75% highest posterior density (HPD) credible intervals for each of the 8 coefficients. These intervals reflect a 90% familywise error rate using a standard Bonferroni correction for multiple comparison. For more information about Bayesian credible and HPD intervals, see [39].

**Table 2:** Parameter estimates for the full model.

Factor	Estimate	98.75% HPD Interval
Percentage forest coverage	4.719	[3.535, 5.828]
Percentage surface water coverage	0.518	[0.230, 0.858]
Elevation	0.058	[0.025, 0.089]
Annual relative humidity	0.005	[-0.001, 0.012]
Annual temperature	-0.037	[-0.053, -0.020]
Annual precipitation	0.011	[-0.048, 0.072]
Population density	-3.442e-5	[-5.545e-5, -1.320e-5]
Median household income	0.001	[-0.002, 0.004]

Table 2 indicates that annual relative humidity, annual precipitation, and median household income are not significant predictors of *B. burgdorferi* seroprevalence because their credible intervals contain 0. Removing combinations of these predictors results in 7 potential reduced models. Each of these potential models was fit, and the only model to have all significant predictors was the model which excluded all three of the predictors listed above. This model was selected as the reduced model, and the results for this model are summarized in Table 3. The posterior medians of the remaining model parameters are *φ* = 0.9404, *ρ* = 0.9997, and *τ*^2^ = 0.5958, and these point estimates validate the claim of strong positive spatial and temporal dependence.

**Table 3:** Parameter estimates for the reduced model.

Factor	Estimate	95% HPD Interval
Percentage forest coverage	4.698	[3.781, 5.629]
Percentage surface water coverage	0.501	[0.244, 0.788]
Elevation	0.052	[0.026, 0.085]
Annual temperature	-0.039	[-0.053, -0.018]
Population density	-3.610e-5	[-5.283e-5, -2.059e-5]

Most of the predictors in the reduced model behave intuitively. The coefficients for percent forest and water coverage are positive, and the coefficient for population density is negative, as one might expect. Note that the coefficient of elevation is positive and the coefficient of annual temperature is negative, which may appear to be counterintuitive, for further discussion see the Discussion section. In order to assess how well the Bayesian spatio-temporal model explains the data, the correlation between the baseline and model estimated prevalences was computed. In particular, a baseline estimate for each county was extracted from Fig 3, and is denoted as ${\tilde{p}}_{s}$, for *s* = 1, …, *S*. A model based estimate for each county was then constructed by averaging over the 5 yearly estimates available from the fitted (reduced) model; i.e., the model estimate for the *s*th county is given by ${\hat{p}}_{s}=5^{-1}\sum_{t=1}^{5}{\hat{p}}_{s}\left(t\right)$, where ${\hat{p}}_{s}\left(t\right)$ is the prevalence estimate resulting from the fitted (reduced) model for the *s*th county during the *t*th year. Let $\tilde{P}$ and $\hat{P}$ denote the collection of baseline and model based estimates, respectively, after removing counties not reporting data. The correlation between these two sets was 0.894 indicating that the Bayesian spatio-temporal model provides a good fit to these data.

### Forecasting

Under the Bayesian spatio-temporal model, forecasting *B. burgdorferi* seroprevalence in domestic dogs is tantamount to forecasting the factor levels and the spatio-temporal random effects. In this section, the methods used to forecast these variables are elucidated. First, since the primary goal of this work is to provide for a one year ahead forecast, it is reaonable to assume that certain risk factors are static; i.e., the current years value can be used as the forecasted value since expected changes are negligible. These variables include, forestation, water coverage, and elevation. Thus, the risk factors that need to be forecasted for each county are annual temperature and population density.

To forecast annual temperature, historical temperature records were collected from 1895 to 2015 for each county and modeled as an AR(1) model. The AR(1) model for an annual temperature series {*F*_*t*_} (previously denoted by {*X*_*s*,1_(*t*)} for county *s* and time *t*) adheres to the following difference equation

$$F_{t}=\delta +\gamma F_{t-1}+\omega_{t},$$

where {*ω*_*t*_} is zero mean white noise. Standard statistical software packages (e.g., R and SAS) can be used to easily fit AR(1) models. Let $\hat{\delta }$ and $\hat{\gamma }$ denote estimates of *δ* and *γ*, respectively, and using these estimates a prediction of the annual temperature at year *t* + 1 from temperatures from year 1 to year *t* is obtained as

$${\hat{F}}_{t+1}=\hat{\delta }+\hat{\gamma }F_{t}.$$

In the proposed forecasting method, ${\hat{F}}_{t+1}$ is used as next year’s annual temperature value. For more information, see [40].

Forecasting the county population density for next year requires the county areas and their recent population counts. The US Census provides reliable county population counts for 2010 and estimated state populations for the years of 1969-2014. A simple linear regression model, with time being the independent variable, was fitted to this state level population data. From this model the county population can be forecasted by first predicting the state population and then partitioning this value into the counties within the state at a proportion that agrees with 2010 Census. The forecasted population density is then obtained by dividing the county population by the county’s area.

To forecast the spatial and temporal random effects a year in advance, Eq (4) is used. In particular, since the ***ϕ***_*t*_’s are independent and identically distributed over various years, given values of *τ*^2^ and *ρ* (available from the posterior samples), ***ϕ***_*t*+1_ is generated randomly from the multivariate normal distribution N(**0**, *τ*^2^(**D** − *ρ* **W**)^−1^). Then ***ξ***_*t*+1_ is set to ***ξ***_*t*+1_ = *φ****ξ***_*t*_ + ***ϕ***_*t*+1_. This process is repeated for each pair of *ρ* and *τ*^2^ available from the posterior sample, thus yielding a sample of the next year’s random effects, for further details see [39, 40].

**Fig 4:**
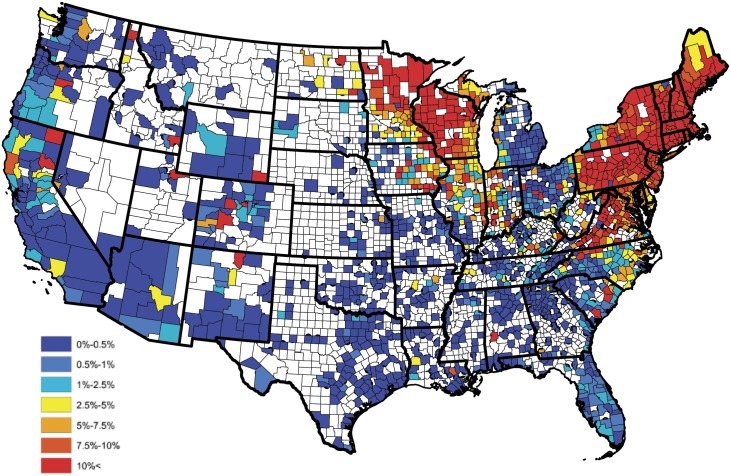
Observed *B. burgdorferi* antibody prevalence in domestic dogs for 2015.

**Fig 5:**
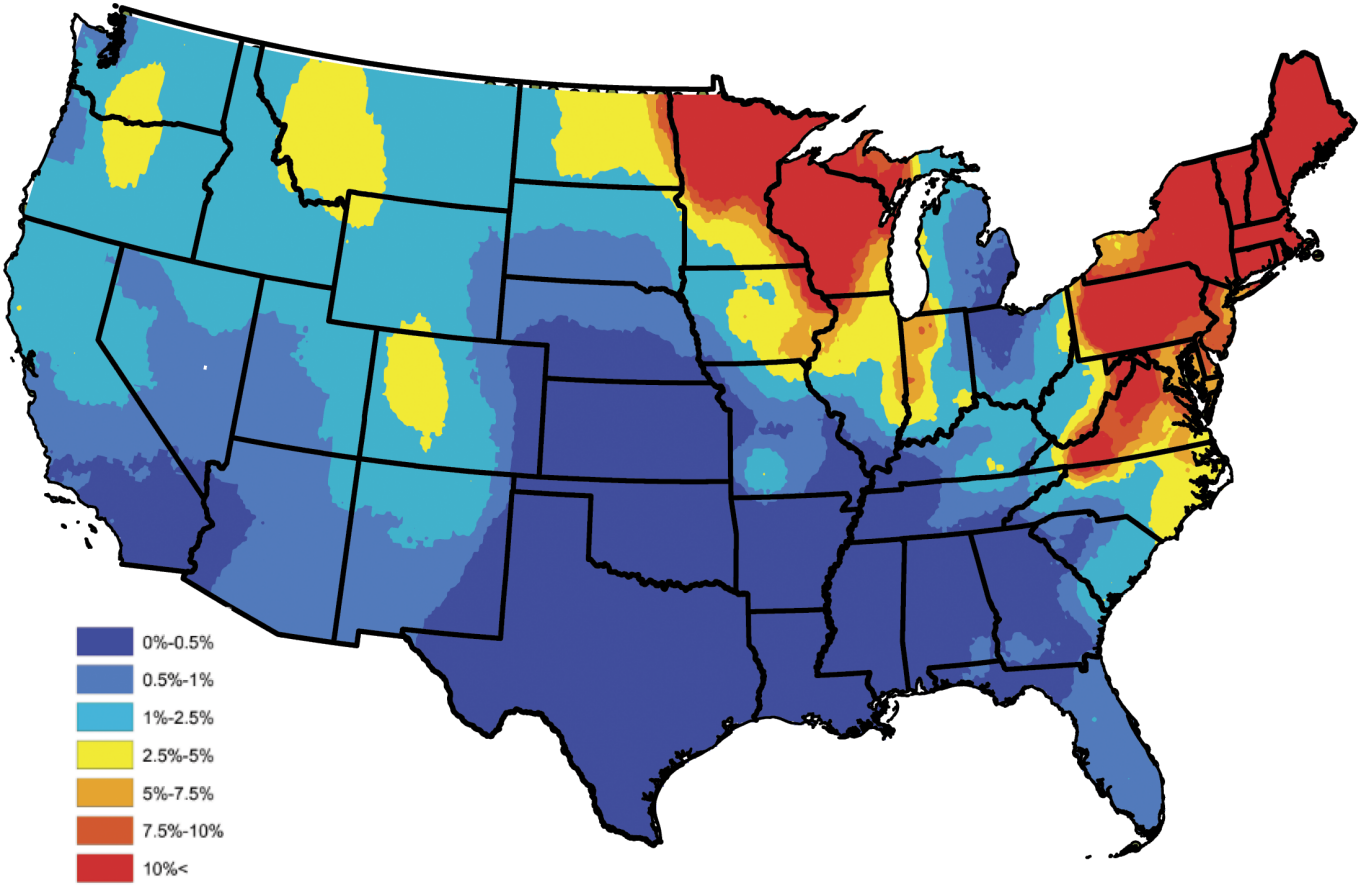
Forecasted *B. burgdorferi* antibody prevalence in domestic dogs for 2015.

**Fig 6:**
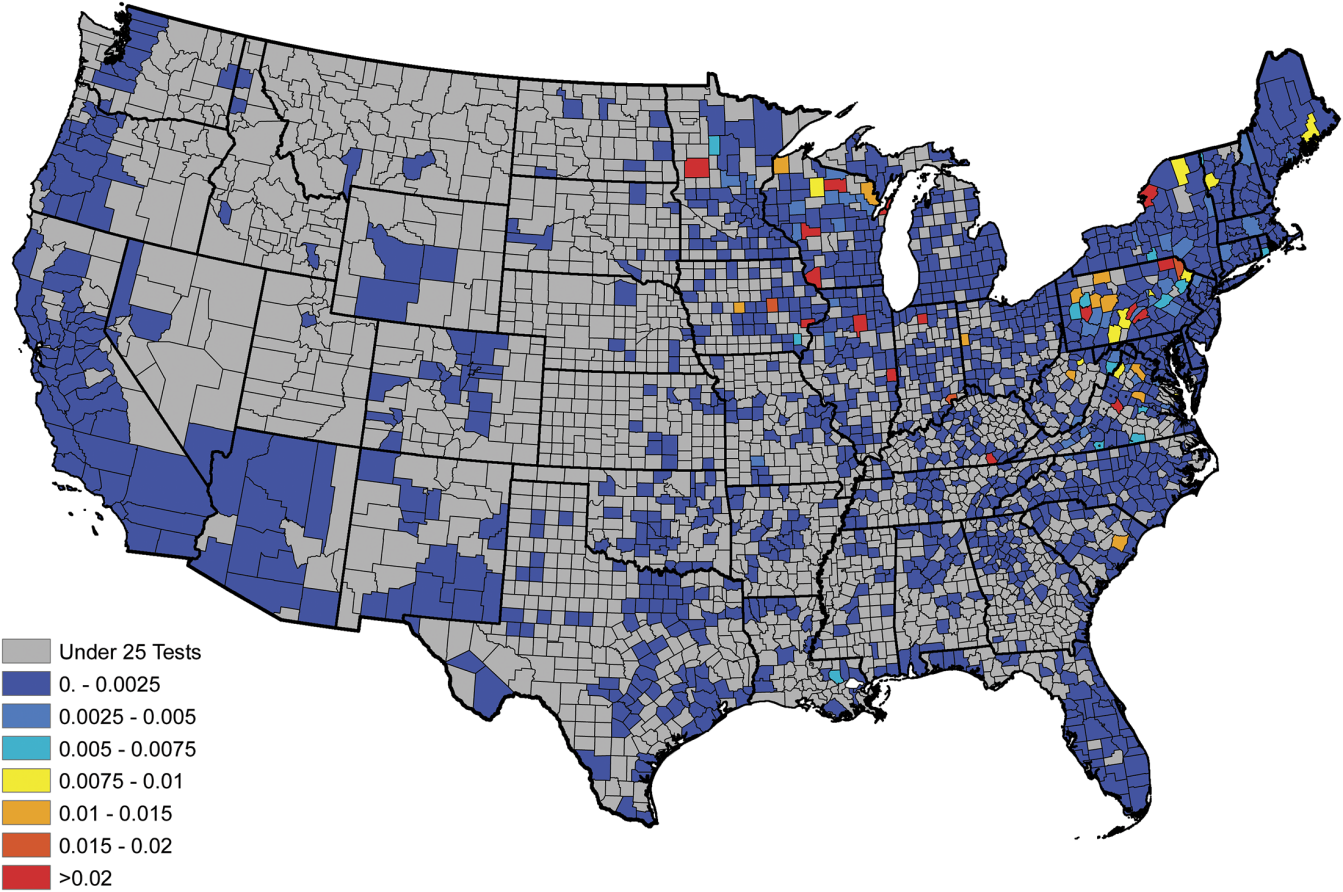
Localized predicitve capacity: Squared difference between the observed and forecasted *B. burgdorferi* seroprevalences for counties reporting more than 25 test results during 2015.

In order to assess the fidelity of the proposed forecasting methods, the 2015 test and factor data were removed and the reduced model was fit to the data collected during the years of 2011-2014. The methods described above were then implemented to forecast the 2015 prevalence of *B. burgdorferi* antibodies in domestic dogs. Figs 4 and 5 present the observed and forecasted *B. burgdorferi* seroprevalences, respectively, for 2015. Further, Fig 6 quantifies the localized predictive capability of the proposed approach by depicting the squared difference between the observed and forecasted *B. burgdorferi* seroprevalences for counties reporting more than 25 test results during 2015. From this figure, one will note that the proposed approach provides an accurate regional forecast throughout the contiguous United States. Note, counties reporting fewer than 25 tests were excluded for the small sample size issues discussed previously. To provide a global assessment of the predictive capacity of the proposed forecasting technique, the weighted correlation (with county weights being set to be the number of tests reported; i.e., *n*_*s*_(*t*)) between the observed and forecasted prevalence estimates was computed, after removing counties not reporting data, with a value of 0.978 being obtained. Thus, this finding tends to suggest that the proposed approach can be used to accurately forecast future trends in *B. burgdorferi* seroprevalence within the contiguous United States. Here the weighted correlation between two sets, say $A={\left\{a_{s}\right\}}_{s=1}^{S}$ and $B={\left\{b_{s}\right\}}_{s=1}^{S}$, is defined as

$$\begin{array}{c} \text{Corr}\left(A,B\right)=\frac{\sum_{s=1}^{S}w_{s}\left(a_{s}-\bar{a}\right)\left(b_{s}-\bar{b}\right)}{\sqrt{\sum_{s=1}^{S}w_{s}{\left(a_{s}-\bar{a}\right)}^{2}\sum_{s=1}^{S}w_{s}{\left(b_{s}-\bar{b}\right)}^{2}}} \end{array}$$ (11)

where *w*_*s*_ denotes the weight assigned to the *s*th observation and

$$\begin{array}{c} \bar{a}=\frac{\sum_{s=1}^{S}w_{s}a_{s}}{\sum_{s=1}^{S}w_{s}}\;\bar{b}=\frac{\sum_{s=1}^{S}w_{s}b_{s}}{\sum_{s=1}^{S}w_{s}}. \end{array}$$

**Fig 7:**
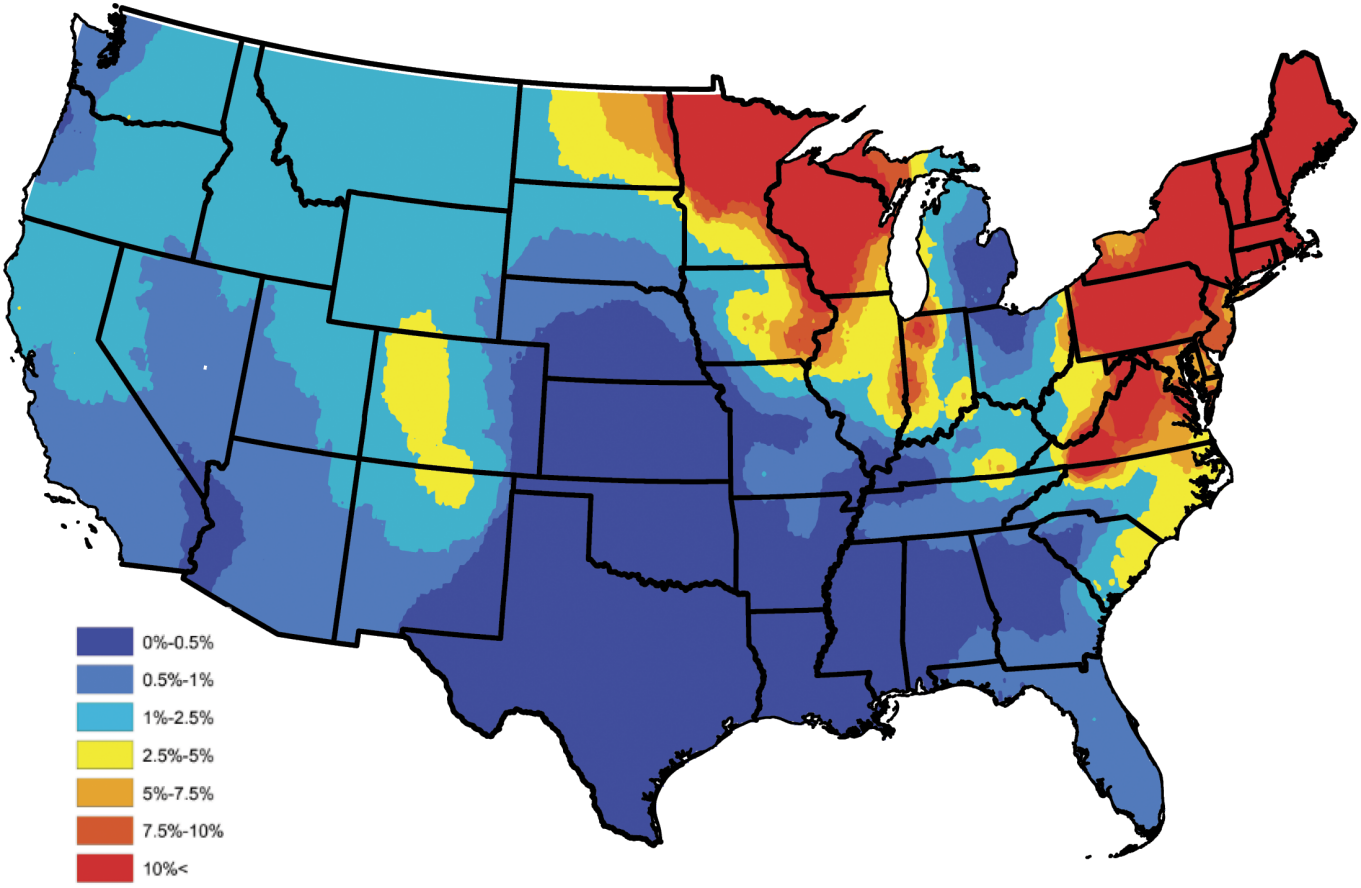
Forecasted *B. burgdorferi* antibody prevalence in domestic dogs for 2016.

Note, the purpose of a weighted correlation is identical to that of the usual correlation, with the exception that it accounts for unequal sample sizes through the weights. Fig 7 presents the 2016 forecast of *B. burgdorferi* prevalence within the contiguous United States, after smoothing (Kriging with default parameters were used in the software ArcGIS).

## Discussion

The objective of this study was to evaluate the space-time patterns and the environmental and socioeconomic drivers of canine *B. burgdorferi* prevalence in the contiguous U.S. using a Bayesian hierarchical modeling approach. Our data were based on serologic testing results for *B. burgdorferi* C6 antigen between 2011-2015. Bayesian hierarchical models have advantages over frequentist approaches for analyzing infectious disease datasets with inherent space-time dependency [37, 58–60], such as clusters of cases that may be linked due to specific environmental drivers. This is particularly relevant for vector-borne disease as the spatial distribution of most vectors is largely determined by environmental and climatologic conditions and the presence of suitable reservoir hosts [1, 61–63]. As such, we included relevant ecological covariates, where available, in our model to help explain variability in our aggregated dataset and to strengthen inferences from our Bayesian spatio-temporal model.

The majority of the predictors in our reduced model of covariates are biologically relevant. The coefficients for percent forest and water coverage are positive, and the coefficient for population density is negative, supporting the established role of vector and vertebrate host ecology in disease transmission [64]. Tick vectors rely on specific environmental and microhabitat factors for survival while off the host [65]. The ecology of *B. burgdorferi* is complex and involves numerous vertebrate species that may serve different roles such as reservoirs, dilution hosts, and hosts for the ticks (i.e., white-tailed deer), and all of these are impacted by habitat and anthropogenic changes [66]. Interestingly, the coefficient of elevation is positive and the coefficient of annual temperature is negative, which is counterintuitive to our understanding of tick ecology and consensus opinion that ticks in the Eastern United States rarely inhabit high elevations (i.e,. Appalachian Mountains) [61–63]. This apparent paradox is likely due to the fact that Lyme disease is more prevalent in the Northeastern United States [34], which compared with the rest of the country, has a relatively low annual temperature and relatively high elevation. It is important to remember that the proposed model relates the mean of the response to the predictors in a linear fashion and is fitted jointly within the range of the observed data. That is, the relationship between the predictors and the prevalence of Lyme disease is only valid for predictor levels within the range of the observed predictor values, with validity declining at the margins. Further, extrapolations to factor levels not in the range of the considered data set will likely lead to contradictions. Thus the positive coefficient of elevation does not imply that Lyme disease prevalence will continue to increase at extreme elevations, nor does the negative coefficient of annual temperature imply that Lyme disease seroprevalence will continue to increase at extremely low temperatures.

Similar to reported incidence of human Lyme disease [67], we observe higher 2011-2015 aggregated *B. burgdorferi* seroprevalence in dogs from Connecticut, Delaware, Maine, Massachusetts, Maryland, Minnesota, New Hampshire, New Jersey, New York, Pennsylvania, Rhode Island, Vermont, Virginia, and Wisconsin. However, in contrast, based on data from dogs, we observed an expansion of this endemic range to include Northern California, Southeastern Oregon, Southwestern Idaho, Eastern Colorado and Northern New Mexico (Fig 1). Perhaps most striking is the recognized expansion of seropositive dogs on the northern border of the contiguous U.S. along the Canadian border, including North Dakota, and the border of Northern Montana and Idaho. The westward expansion of canine *B. burgdorferi* seroprevalence from Minnesota into North Dakota mirrors recent reports that Lyme disease is poised to be a significant human public health concern in North Dakota [68]. Further, this observation supports and extends recent concern over northward expansion of *B. burgdorferi* infected ticks into Canada from the Northeastern and Midwestern United States [69, 70].

From five years of historical diagnostic tests, our data show that a Bayesian model can capably quantify *B. burgdorferi* seroprevalence, which ultimately will support qualitative decision-making and surveillance in disease management and response. When comparing actual to forecasted *B. burgdorferi* seroprevalence in 2015, a weighted correlation of 0.978 was achieved, demonstrating significant predictive skill. Finally, using our predictive forecast model we report forecasted 2016 canine *B. burgdorferi* seroprevalence. Of note is the apparent convergence of *B. burgdorferi* infection of dogs from the Northeastern and Mid-Central United States in the Great Lakes region, encompassing Indiana, Ohio, Illinois, Kentucky and Michigan. This observation is supported by recent reports of encroaching *I. scapularis* into this region [71], and suggests annual testing of dogs in these states, as well as North Dakota and bordering Canadian provinces is strongly warranted. As an adjunct to annual testing, year-round use of acaracides in dogs can reduce tick infestation, thereby reducing the potential for not only *B. burgdorferi* transmission, but also several other important canine tick-borne pathogens [72, 73]. Finally, vaccination of dogs against Lyme disease to prevent *B. burgdorferi* infection has been shown to be effective in controlled experimental infection studies [74–77] and in protection against natural infection in dogs living in endemic areas [78, 79]. In endemic areas, dogs that are vaccine protocol-compliant are significantly less likely to become infected with *B. burgdorferi* [80]. Vaccine studies have concluded that emphasis should be placed on vaccinating dogs at risk for Lyme disease before they are exposed to infected ticks [79]. As such, we suggest the forecasted areas with an increased likelihood of *B. burgdorferi* transmission outside of the established endemic areas should provide veterinary practitioners with evidence-based options for recommendation of Lyme disease vaccines to clients and protection of dogs against emerging disease.

Limitations to this study include selection bias of the canine population. As mentioned above, samples were submitted for testing by veterinary clinics, and therefore represent dogs under the care of a veterinarian. This suggests that the data are a conservative estimate of the prevalence in domestic dogs because dogs at the highest risk of tick exposure would be those that receive no veterinary care, those from lower socioeconomic families, or are owned by clients who decline these additional tests during wellness visits. Additionally, a lack of knowledge about the distribution of tick-borne pathogens may limit the testing that veterinarians request; however, these tests are often run during routine heartworm testing so this latter issue is likely a minimal concern. Additional bias could be introduced through variations by region in the use of the diagnostic tests (i.e. whether it is used predominately for wellness visits or for cases in which the disease is suspected prior to testing) or variations by region in the use of reference laboratory services and products. It is also recognized that travel history is not controlled for and will account for some of the cases outside endemic regions. Despite these limitations, these data are acquired on a monthly basis [6], and thus provide a robust and timely source of information about the dynamic change of canine *B. burgdorferi* seroprevalence across the contiguous US, and holds promise for longitudinal studies to better understand the dynamic nature of vector-borne disease over time. There is also evidence that other tick vectors are involved in sustaining the enzootic cycle of *B. burgdorferi ss*, such as *Ixodes affinis*, [81] and so it is important when interpreting these results to consider the possibility of other vectors of *B. burgdorferi*. However, *I. affinis* is believed to be uncommonly found on dogs and does not feed on humans so its impact is considered to be minimal.

Though the proposed technique could be used to construct long-term forecasts, caution should be taken. In particular, our approach makes use of forecasted values of the significant factors, with some factors being assumed to be static throughout time (e.g., forestation and surface water coverage). This assumption is reasonable in the short-term, but would obviously be problematic over a much larger time span; e.g., twenty to fifty years. Moreover, in general, when forecasting future trends one should be cautious of long-term forecasts, due to possible violations of assumed model forms not apparent in the available data; e.g., changes in population density that may be spatially variable throughout time. Thus, we promote the use of our approach to provide only short-term forecasts of spatial trends in *B. burgdorferi* seroprevalence.

Similar to domestic dogs, spatial risk models for human Lyme disease are needed to address the rise in human Lyme disease incidence. Ideally, spatial risk models for human Lyme disease would be based on vector abundance inclusive of pathogen burden [82, 83]. In comparison to other vector-borne diseases, Lyme disease risk assessment is facilitated by the observation that *B. burgdorferi* prevalence in *Ixodes* spp. from Lyme disease endemic regions is relatively high [82, 84, 85]. Canine *B. burgdorferi* seroprevalence has been cited as one potential tool for Lyme disease risk assessment in humans [44, 86]. As noted by Mead et al. [44], canine *B. burgdorferi* seroprevalence greater than 5% at the county level was associated with human risk of Lyme disease [44], while less than 1% canine seroprevalence was associated with little to no risk for human cases of Lyme disease [86]. We further this suggestion that the use of canine *B. burgdorferi* seropositivity has merit as a risk assessment tool in both endemic and non-endemic regions. In particular, areas where *B. burgdorferi* seroprevalence is greater than 1%, but increases over time, may be an area to focus tick-prevention messages as these areas may be at an increased risk for human Lyme disease. As such, we believe that with further research into the relationship between human and canine Lyme disease, canine *B. burgdorferi* seropositivity has the potential to function as an early warning system for geographic expansion and emerging infection risk in humans [17], and support targeted vector surveillance efforts to monitor *Ixodes* spp. *B. burgdorferi* infection in a cost-effective manner. As the increasing incidence of Lyme disease continues to put pressure on the United States healthcare system [7], the use of canine *B. burgdorferi* seroprevalence to ultimately forecast spatial and temporal patterns of risk of human Lyme disease is a promising tool for targeting public health educational campaigns and resources for vector surveillance to best protect humans and veterinary species from disease.

## References

[1] Mead PS. Epidemiology of Lyme disease. Infect Dis Clin North Am. 2015;29(2):187–210. 10.1016/j.idc.2015.02.010 25999219

[2] Stanek G, Wormser GP, Gray J, Strle F. Lyme borreliosis. Lancet. 2012;379(9814):461–473. 10.1016/S0140-6736(11)60103-7 21903253

[3] Steere AC, Coburn J, Glickstein L. The emergence of Lyme disease. J Clin Invest. 2004;113(8):1093–1101. 10.1172/JCI21681 15085185 PMC385417

[4] Nelson CA, Saha S, Kugeler KJ, Delorey MJ, Shankar MB, Hinckley AF, et al Incidence of Clinician-Diagnosed Lyme Disease, United States, 2005-2010. Emerg Infect Dis. 2015;21(9):1625–1631. 10.3201/eid2109.150417 26291194 PMC4550147

[5] Hinckley AF, Connally NP, Meek JI, Johnson BJ, Kemperman MM, Feldman KA, et al Lyme disease testing by large commercial laboratories in the United States. Clin Infect Dis. 2014;59(5):676–81. 10.1093/cid/ciu397 24879782 PMC4646413

[6] Companion Animal Parasite Council; 2012-current. Available from: www.CAPCvet.org.

[7] Adrion ER, Aucott J, Lemke KW, Weiner JP. Health care costs, utilization and patterns of care following Lyme disease. PLoS One. 2015;10(2):e0116767 10.1371/journal.pone.0116767 25650808 PMC4317177

[8] McNabb SJ, Jajosky RA, Hall-Baker PA, Adams DA, Sharp P, Anderson WJ, et al Summary of notifiable diseases—United States, 2005. MMWR Morb Mortal Wkly Rep. 2007;54(53):1–92. 17392681

[9] Wormser GP, Dattwyler RJ, Shapiro ED, Halperin JJ, Steere AC, Klempner MS, et al The clinical assessment, treatment, and prevention of lyme disease, human granulocytic anaplasmosis, and babesiosis: clinical practice guidelines by the Infectious Diseases Society of America. Clin Infect Dis. 2006;43(9):1089–1134. 10.1086/508667 17029130

[10] Littman MP, Goldstein RE, Labato MA, Lappin MR, Moore GE. ACVIM small animal consensus statement on Lyme disease in dogs: diagnosis, treatment, and prevention. J Vet Intern Med. 2006;20(2):422–34. 10.1111/j.1939-1676.2006.tb02880.x 16594606

[11] Steere AC, Dhar A, Hernandez J, Fischer PA, Sikand VK, Schoen RT, et al Systemic symptoms without erythema migrans as the presenting picture of early Lyme disease. Am J Med. 2003;114(1):58–62. 10.1016/S0002-9343(02)01440-7 12543291

[12] Cohn KA, Thompson AD, Shah SS, Hines EM, Lyons TW, Welsh EJ, et al Validation of a clinical prediction rule to distinguish Lyme meningitis from aseptic meningitis. Pediatrics. 2012;129(1):e46–53. 10.1542/peds.2011-1215 22184651

[13] Costello JM, Alexander ME, Greco KM, Perez-Atayde AR, Laussen PC. Lyme carditis in children: presentation, predictive factors, and clinical course. Pediatrics. 2009;123(5):e835–841. 10.1542/peds.2008-3058 19403477

[14] Arvikar SL, Steere AC. Diagnosis and treatment of Lyme arthritis. Infect Dis Clin North Am. 2015;29(2):269–280. 10.1016/j.idc.2015.02.004 25999223 PMC4443866

[15] Thompson A, Mannix R, Bachur R. Acute pediatric monoarticular arthritis: distinguishing lyme arthritis from other etiologies. Pediatrics. 2009;123(3):959–65. 10.1542/peds.2008-1511 19255026

[16] Bachur RG, Adams CM, Monuteaux MC. Evaluating the child with acute hip pain (“irritable hip”) in a Lyme endemic region. J Pediatr. 2015;166(2):407–411 e1. 10.1016/j.jpeds.2014.09.040 25444013

[17] Little SE, Heise SR, Blagburn BL, Callister SM, Mead PS. Lyme borreliosis in dogs and humans in the USA. Trends Parasitol. 2010;26(4):213–218. 10.1016/j.pt.2010.01.006 20207198

[18] Detmer SE, Bouljihad M, Hayden DW, Schefers JM, Armien A, Wunschmann A. Fatal pyogranulomatous myocarditis in 10 Boxer puppies. J Vet Diagn Invest. 2016;28(2):144–149. 10.1177/1040638715626486 26965234

[19] Krupka I, Straubinger RK. Lyme borreliosis in dogs and cats: background, diagnosis, treatment and prevention of infections with *Borrelia burgdorferi* sensu stricto. Vet Clin North Am Small Anim Pract. 2010;40(6):1103–19. 10.1016/j.cvsm.2010.07.011 20933139

[20] Wiseman-Orr ML, Nolan AM, Reid J, Scott EM. Development of a questionnaire to measure the effects of chronic pain on health-related quality of life in dogs. Am J Vet Res. 2004;65(8):1077–1084. 10.2460/ajvr.2004.65.1077 15334841

[21] Mathews K, Kronen PW, Lascelles D, Nolan A, Robertson S, Steagall PV, et al Guidelines for recognition, assessment and treatment of pain: WSAVA Global Pain Council members and co-authors of this document. J Small Anim Pract. 2014;55(6):E10–68. 10.1111/jsap.12200 24841489

[22] Benito J, Depuy V, Hardie E, Zamprogno H, Thomson A, Simpson W, et al Reliability and discriminatory testing of a client-based metrology instrument, feline musculoskeletal pain index (FMPI) for the evaluation of degenerative joint disease-associated pain in cats. Vet J. 2013;196(3):368–373. 10.1016/j.tvjl.2012.12.015 23369382

[23] Branda JA, Aguero-Rosenfeld ME, Ferraro MJ, Johnson BJ, Wormser GP, Steere AC. 2-tiered antibody testing for early and late Lyme disease using only an immunoglobulin G blot with the addition of a VlsE band as the second-tier test. Clin Infect Dis. 2010;50(1):20–26. 10.1086/648674 19947857

[24] Lipsett SC, Pollock NR, Branda JA, Gordon CD, Gordon CR, Lantos PM, et al The Positive Predictive Value of Lyme Elisa for the Diagnosis of Lyme Disease in Children. Pediatr Infect Dis J. 2015;34(11):1260–1262. 10.1097/INF.0000000000000858 26222064 PMC4630803

[25] Stillman BA, Monn M, Liu J, Thatcher B, Foster P, Andrews B, et al Performance of a commercially available in-clinic ELISA for detection of antibodies against *Anaplasma phagocytophilum*, *Anaplasma platys*, *Borrelia burgdorferi*, *Ehrlichia canis*, and *Ehrlichia ewingii* and *Dirofilaria immitis* antigen in dogs. J Am Vet Med Assoc. 2014;245(1):80–86. 10.2460/javma.245.1.80 24941391

[26] Bacon RM, Biggerstaff BJ, Schriefer ME, Gilmore RD Jr, Philipp MT, Steere AC, et al Serodiagnosis of Lyme disease by kinetic enzyme-linked immunosorbent assay using recombinant VlsE1 or peptide antigens of *Borrelia burgdorferi* compared with 2-tiered testing using whole-cell lysates. J Infect Dis. 2003;187(8):1187–1199. 10.1086/374395 12695997 PMC7109709

[27] Embers ME, Hasenkampf NR, Barnes MB, Didier ES, Philipp MT, Tardo AC. Five-Antigen Fluorescent Bead-Based Assay for Diagnosis of Lyme Disease. Clin Vaccine Immunol. 2016;23(4):294–303. 10.1128/CVI.00685-15 26843487 PMC4820514

[28] Nyman D, Willen L, Jansson C, Carlsson SA, Granlund H, Wahlberg P. VlsE C6 peptide and IgG ELISA antibody analysis for clinical diagnosis of Lyme borreliosis in an endemic area. Clin Microbiol Infect. 2006;12(5):496–497. 10.1111/j.1469-0691.2006.01374.x 16643532

[29] Wagner B, Freer H, Rollins A, Garcia-Tapia D, Erb HN, Earnhart C, et al Antibodies to *Borrelia burgdorferi* OspA, OspC, OspF, and C6 antigens as markers for early and late infection in dogs. Clin Vaccine Immunol. 2012;19(4):527–535. 10.1128/CVI.05653-11 22336289 PMC3318275

[30] Little SE, Beall MJ, Bowman DD, Chandrashekar R, Stamaris J. Canine infection with *Dirofilaria immitis*, *Borrelia burgdorferi*, *Anaplasma* spp., and *Ehrlichia* spp. in the United States, 2010-2012. Parasit Vectors. 2014;7:257 10.1186/1756-3305-7-257 24886589 PMC4057565

[31] Bowman D, Little SE, Lorentzen L, Shields J, Sullivan MP, Carlin EP. Prevalence and geographic distribution of *Dirofilaria immitis*, *Borrelia burgdorferi*, *Ehrlichia canis*, and *Anaplasma phagocytophilum* in dogs in the United States: results of a national clinic-based serologic survey. Vet Parasitol. 2009;160(1-2):138–48. 10.1016/j.vetpar.2008.10.093 19150176

[32] Scott JD. Studies abound on how far north *Ixodes scapularis* ticks are transported by birds. Ticks and Tick-borne Diseases. 2016;7:327–328. 10.1016/j.ttbdis.2015.12.001 26739029

[33] Madhav NK, Brownstein JS, Tsao JI, Fish D. A Dispersal Model for the Range Expansion of Blacklegged Tick (Acari: Ixodidae). Med Entomol. 2004;41(5):842–852. 10.1603/0022-2585-41.5.842 15535611

[34] Adams DA, Jajosky RA, Ajani U, Kriseman J, Sharp P, Onwen DH, et al Summary of notifiable diseases–United States, 2012. MMWR Morb Mortal Wkly Rep. 2014;61(53):1–121. 25233134

[35] Centers for Disease Control and Prevention; 2015. Available from: http://www.cdc.gov/lyme/stats/index.html

[36] Eisen RJ, Eisen L, Beard CB. County-Scale Distribution of *Ixodes scapularis* and *Ixodes pacificus* (Acari: Ixodidae) in the Continental United States. J Med Entomol. 2016; 10.1093/jme/tjv237 26783367 PMC4844559

[37] Bowman DD, Liu Y, McMahan CS, Nordone SK, Yabsley MJ, Lund RB. Forecasting United States heartworm *Dirofilaria immitis* prevalence in dogs. Parasit Vectors. 2016;9(1):540 10.1186/s13071-016-1804-y 27724981 PMC5057216

[38] Liu Y, Lund RB, Nordone SK, Yabsley MJ, McMahan CS. A Bayesian spatio-temporal model for forecasting the prevalence of antibodies to *Ehrlichia* species in domestic dogs within the contiguous United States. Parasit Vectors. 2016;In Press. 10.1186/s13071-017-2068-x PMC5343545 28274248

[39] Gelman A, Carlin JB, Stern HS, Dunson DB, Vehtari A, Rubin DB. Bayesian Data Analysis. 3rd ed Boca Raton, FL: Chapman & Hall/CRC; 2014.

[40] Harrison J, West M. Bayesian Forecasting and Dynamic Models. New York City: Springer; 1999.

[41] Besag J, Mondal D. Exact goodness-of-fit tests for Markov chains. Biometrics. 2013;69(2):488–496. 10.1111/biom.12009 23432148

[42] Hosking FJ, Sterne JA, Smith GD, Green PJ. Inference from genome-wide association studies using a novel Markov model. Genet Epidemiol. 2008;32(6):497–504. 10.1002/gepi.20322 18383184

[43] Stich RW, Blagburn BL, Bowman DD, Carpenter C, Cortinas MR, Ewing SA, et al Quantitative factors proposed to influence the prevalence of canine tick-borne disease agents in the United States. Parasit Vectors. 2014;7:417 10.1186/1756-3305-7-417 25185829 PMC4167287

[44] Mead P, Goel R, Kugeler K. Canine serology as adjunct to human Lyme disease surveillance. Emerg Infect Dis. 2011;17(9):1710–2. 10.3201/1709.110210 21888800 PMC3322085

[45] Levy SA, O’Connor TP, Hanscom JL, Shields P, Lorentzen L, Dimarco AA. Quantitative measurement of C6 antibody following antibiotic treatment of *Borrelia burgdorferi* antibody-positive nonclinical dogs. Clinical and vaccine immunology: CVI. 2008;15(1):115–9. 10.1128/CVI.00340-07 18003819 PMC2223868

[46] Ting Liang F, Jacobson RH, Straubinger RK, Grooters A, Philipp MT. Characterization of a Borrelia burgdorferi VlsE Invariable Region Useful in Canine Lyme Disease Serodiagnosis by Enzyme-Linked Immunosorbent Assay. Journal of Clinical Microbiology. 2000;38(11):4160–4166. 11060084 10.1128/jcm.38.11.4160-4166.2000 PMC87557

[47] IDEXX Laboratories, Inc;. Available from: http://www.idexx.com/.

[48] Wang D, Bowman DD, Brown HE, Harrington LC, Kaufman PE, McKay T, et al Factors influencing U.S. canine heartworm (*Dirofilaria immitis*) prevalence. Parasit Vectors. 2014;7:264 10.1186/1756-3305-7-264 24906567 PMC4101712

[49] Environmental Systems Research Institute I. Documentation; 2016. Available from: http://desktop.arcgis.com/en/documentation/.

[50] Banerjee S, Carlin BP, Gelfand AE. Hierarchical Modeling and Analysis for Spatial Data. 2nd ed Boca Raton, FL: Chapman and Hall/CRC; 2014.

[51] Besag J. Spatial interaction and the statistical analysis of lattice systems. Journal of the Royal Statistical Society Series B. 1974;36:192–236.

[52] Lopez-Quilez A, Munoz F. Review of spatio-temporal models for disease mapping. Final report for the Euroheis 2 project. Zenodo.; 2009. Available from: http://www.uv.es/~famarmu/doc/Euroheis2-report.pdf.

[53] Martinez-Beneito MA, Lopez-Quilez A, Botella-Rocamora P. An autoregressive approach to spatio-temporal disease mapping. Stat Med. 2008;27(15):2874–89. 10.1002/sim.3103 17979141

[54] Nobre AA, Schmidt AM, Lopes HF. Spatio-temporal models for mapping the incidence of malaria in Pará. Environmetrics. 2005;16(3):291–304. 10.1002/env.704

[55] Xia H, Carlin BP. Spatio-temporal models with errors in covariates: mapping Ohio lung cancer mortality. Stat Med. 1998;17(18):2025–43. 10.1002/(SICI)1097-0258(19980930)17:18<2025::AID-SIM865>3.0.CO;2-M 9789912

[56] Cressie N, Wikle CK. Statistics for spatio-temporal data Wiley series in probability and statistics. Hoboken, N.J.: Wiley; 2011.

[57] Brockwell PJ, Davis RA. In: Introduction to Time Series and Forecasting. New York City: Springer-Verlag; 2002 10.1007/b97391

[58] Hanzlicek GA, Raghavan RK, Ganta RR, Anderson GA. Bayesian Space-Time Patterns and Climatic Determinants of Bovine Anaplasmosis. PLoS One. 2016;11(3):e0151924 10.1371/journal.pone.0151924 27003596 PMC4803217

[59] Raghavan RK, Neises D, Goodin DG, Andresen DA, Ganta RR. Bayesian spatio-temporal analysis and geospatial risk factors of human monocytic ehrlichiosis. PLoS One. 2014;9(7):e100850 10.1371/journal.pone.0100850 24992684 PMC4081574

[60] Raghavan RK, Goodin DG, Neises D, Anderson GA, Ganta RR. Hierarchical Bayesian Spatio-Temporal Analysis of Climatic and Socio-Economic Determinants of Rocky Mountain Spotted Fever. PLoS One. 2016;11(3):e0150180 10.1371/journal.pone.0150180 26942604 PMC4778859

[61] Nieto NC, Holmes EA, Foley JE. Survival rates of immature *Ixodes pacificus* (Acari: Ixodidae) ticks estimated using field-placed enclosures. J Vector Ecol. 2010;35(1):43–9. 10.1111/j.1948-7134.2010.00056.x 20618646

[62] Ogden NH, Mechai S, Margos G. Changing geographic ranges of ticks and tick-borne pathogens: drivers, mechanisms and consequences for pathogen diversity. Front Cell Infect Microbiol. 2013;3:46 10.3389/fcimb.2013.00046 24010124 PMC3756306

[63] Ogden NH, Radojevic M, Wu X, Duvvuri VR, Leighton PA, Wu J. Estimated effects of projected climate change on the basic reproductive number of the Lyme disease vector *Ixodes scapularis*. Environ Health Perspect. 2014;122(6):631–638. 10.1289/ehp.1307799 24627295 PMC4050516

[64] Ogden NH, Bigras-Poulin M, O’Callaghan CJ, Barker IK, Kurtenbach K, Lindsay LR, et al Vector seasonality, host infection dynamics and fitness of pathogens transmitted by the tick *Ixodes scapularis*. Parasitology. 2007;134(Pt 2):209–227. 10.1017/S0031182006001417 17032476

[65] Parham PE, Waldock J, Christophides GK, Hemming D, Agusto F, Evans KJ, et al Climate, environmental and socio-economic change: weighing up the balance in vector-borne disease transmission. Philos Trans R Soc Lond B Biol Sci. 2015;370 (1665). 10.1098/rstb.2013.0551 25688012 PMC4342957

[66] Levi T, Keesing F, Holt RD, Barfield M, Ostfeld RS. Quantifying dilution and amplification in a community of hosts for tick-borne pathogens. Ecol Appl. 2016;28(2):484–498. 10.1890/15-0122 27209790

[67] Adams DA, Gallagher KM, Jajosky RA, Kriseman J, Sharp P, Anderson WJ, et al Summary of Notifiable Diseases—United States, 2011. MMWR Morb Mortal Wkly Rep. 2013;60(53):1–117. 23820934

[68] Stone BL, Russart NM, Gaultney RA, Floden AM, Vaughan JA, Brissette CA. The Western progression of lyme disease: infectious and nonclonal *Borrelia burgdorferi* Sensu Lato populations in Grand Forks County, North Dakota. Appl Environ Microbiol. 2015;81(1):48–58. 10.1128/AEM.02422-14 25304515 PMC4272707

[69] Gasmi S, Ogden NH, Leighton PA, Lindsay LR, Thivierge K. Analysis of the human population bitten by *Ixodes scapularis* ticks in Quebec, Canada: Increasing risk of Lyme disease. Ticks Tick Borne Dis. 2016;7(6):1075–1081. 10.1016/j.ttbdis.2016.09.006 27650641

[70] Nelder MP, Russell C, Lindsay LR, Dhar B, Patel SN, Johnson S, et al Population-based passive tick surveillance and detection of expanding foci of blacklegged ticks *Ixodes scapularis* and the Lyme disease agent *Borrelia burgdorferi* in Ontario, Canada. PLoS One. 2014;9(8):e105358 10.1371/journal.pone.0105358 25171252 PMC4149368

[71] Eisen RJ, Eisen L, Ogden NH, Beard CB. Linkages of Weather and Climate With *Ixodes scapularis* and *Ixodes pacificus* (Acari: Ixodidae), Enzootic Transmission of *Borrelia burgdorferi*, and Lyme Disease in North America. J Med Entomol. 2015; 10.1093/jme/tjv199 26681789 PMC4844560

[72] Spencer JA, Butler JM, Stafford KC, Pough MB, Levy SA, Bledsoe DL, et al Evaluation of permethrin and imidacloprid for prevention of *Borrelia burgdorferi* transmission from blacklegged ticks (*Ixodes scapularis*) to *Borrelia burgdorferi*-free dogs. Parasitol Res. 2003;90(3):S106–7. 10.1007/s00436-003-0904-8 12928869

[73] Wengenmayer C, Williams H, Zschiesche E, Moritz A, Langenstein J, Roepke RK, et al The speed of kill of fluralaner (Bravecto) against *Ixodes ricinus* ticks on dogs. Parasit Vectors. 2014;7:525 10.1186/s13071-014-0525-3 25406442 PMC4247686

[74] LaFleur RL, Dant JC, Wasmoen TL, Callister SM, Jobe DA, Lovrich SD, et al Bacterin that induces anti-OspA and anti-OspC borreliacidal antibodies provides a high level of protection against canine Lyme disease. Clin Vaccine Immunol. 2009;16(2):253–9. 10.1128/CVI.00373-08 19052162 PMC2643534

[75] Rhodes DV, Earnhart CG, Mather TN, Meeus PF, Marconi RT. Identification of *Borrelia burgdorferi* ospC genotypes in canine tissue following tick infestation: implications for Lyme disease vaccine and diagnostic assay design. Vet J. 2013;198(2):412–8. 10.1016/j.tvjl.2013.07.019 23962611 PMC3872846

[76] Conlon JA, Mather TN, Tanner P, Gallo G, Jacobson RH. Efficacy of a nonadjuvanted, outer surface protein A, recombinant vaccine in dogs after challenge by ticks naturally infected with *Borrelia burgdorferi*. Vet Ther. 2000;1:96–107. 19757556

[77] Zoetis. Data on file, Study Report No. B865R-US-12-018, Zoetis Inc; 2016. Available from: https://www.zoetisus.com/products/dogs/vanguard-crlyme/img/assets/crlyme_efficacy_study.pdf.

[78] Levy SA, Lissman BA, Ficke CM. Performance of a *Borrelia burgdorferi* bacterin in borreliosis-endemic areas. J Am Vet Med Assoc. 1993;202(11):1834–8. 8320151

[79] Levy SA. Use of a C6 ELISA test to evaluate the efficacy of a whole-cell bacterin for the prevention of naturally transmitted canine *Borrelia burgdorferi* infection. Vet Ther. 2002;3(4):420–424. 12584679

[80] Eschner AK, Mugnai K. Immunization with a recombinant subunit OspA vaccine markedly impacts the rate of newly acquired *Borrelia burgdorferi* infections in client-owned dogs living in a coastal community in Maine, USA. Parasit Vectors. 2015;8:92 10.1186/s13071-015-0676-x 25890386 PMC4326332

[81] Maggi RG, Reichelt S, Toliver M, Engber B. *Borrelia* species in *Ixodes affinis* and *Ixodes scapularis* ticks collected from the coastal plain of North Carolina. Ticks and Tick-borne Diseases. 2010;1(4):168–171. 10.1016/j.ttbdis.2010.08.003 21771524

[82] Eisen RJ, Eisen L. Spatial modeling of human risk of exposure to vector-borne pathogens based on epidemiological versus arthropod vector data. J Med Entomol. 2008;45(2):181–192. 10.1093/jmedent/45.2.181 18402133

[83] Daniels TJ, Boccia TM, Varde S, Marcus J, Le J, Bucher DJ, et al Geographic risk for lyme disease and human granulocytic ehrlichiosis in southern New York state. Appl Environ Microbiol. 1998;64(12):4663–9. 9835546 10.1128/aem.64.12.4663-4669.1998 PMC90906

[84] Scott JD, Foley JE, Clark KL, Anderson JF, Durden LA, Manord JM, et al Established Population of Blacklegged Ticks with High Infection Prevalence for the Lyme Disease Bacterium, *Borrelia burgdorferi* Sensu Lato, on Corkscrew Island, Kenora District, Ontario. Int J Med Sci. 2016;13(11):881–891. 10.7150/ijms.16922 27877080 PMC5118759

[85] Hutchinson ML, Strohecker MD, Simmons TW, Kyle AD, Helwig MW. Prevalence Rates of *Borrelia burgdorferi* (Spirochaetales: Spirochaetaceae), *Anaplasma phagocytophilum* (Rickettsiales: Anaplasmataceae), and *Babesia microti* (Piroplasmida: Babesiidae) in Host-Seeking *Ixodes scapularis* (Acari: Ixodidae) from Pennsylvania. J Med Entomol. 2015;52(4):693–8. 10.1093/jme/tjv037 26335476

[86] Millen K, Kugeler KJ, Hinckley AF, Lawaczeck EW, Mead PS. Elevated Lyme disease seroprevalence among dogs in a nonendemic county: harbinger or artifact? Vector Borne Zoonotic Dis. 2013;13(5):340–341. 10.1089/vbz.2012.1025 23421882 PMC4703038

